# Host-Pathogen Interaction and Resistance Mechanisms in Dermatophytes

**DOI:** 10.3390/pathogens13080657

**Published:** 2024-08-04

**Authors:** Eleonora Dubljanin, Jelena Zunic, Isidora Vujcic, Ivana Colovic Calovski, Sandra Sipetic Grujicic, Stefan Mijatovic, Aleksandar Dzamic

**Affiliations:** 1 Faculty of Medicine, Institute of Microbiology and Immunology, University of Belgrade, 11000 Belgrade, Serbia; 2 Faculty of Medicine, University of Belgrade, 11000 Belgrade, Serbia; 3 Faculty of Medicine, Institute of Epidemiology, University of Belgrade, 11000 Belgrade, Serbia

**Keywords:** dermatophytes, pathogenesis, virulence factors, immune response, antifungal resistance

## Abstract

Dermatophytes are widely distributed in the environment, with an estimated prevalence of 20–25% of the the global population yearly. These fungi are keratinophilic and keratinolytic and cause the infection of keratin-rich structures such as skin, hair, and nails. The pattern of this infectious disease covers a wide spectrum from exposed individuals without symptoms to those with acutely inflammatory or non-inflammatory, chronic to invasive, and even life-threatening symptoms. This review summarizes current information on the pathogenicity, virulence factors, and drug resistance mechanisms associated with dermatophytes. A greater number of virulence factors of these fungi are important for the occurrence of infection and the changes that occur, including those regarding adhesins, the sulfite efflux pump, and proteolytic enzymes. Other virulence factors include mechanisms of evading the host defense, while the development of resistance to antifungal drugs is increasing, resulting in treatment failure. The investigation of host-pathogen interactions is essential for developing a more complete understanding of the mechanisms underlying dermatophyte pathogenesis and host response to inform the use of diagnostics methods and antifungal therapeutics to minimize the high fungal burden caused by dermatophytes and to control the spread of resistance.

## 1. Introduction

Dermatophytes represent a group of related species of filamentous fungi distributed in three well-known genera: *Trichophyton* spp., *Epidermophyton* sp., and *Microsporum* spp. However, recent taxonomic advances have broadened the classification of dermatophytes, with the introduction of the additional genera *Nannizzia, Arthroderma, Paraphyton, Lophophyton, Ctenomyces, and Guarromyces* [1]. These fungi are keratinophilic and keratinolytic and cause the infection of keratin-rich structures such as skin, hair, and nails [2]. The fungal nature of dermatophytosis was recognized for the first time in 1843 when Gruby described *Microsporum audouinii* as the causative agent of tinea capitis [3], based on the clinical characteristics of the disease and the appearance of the fungi in microscopic preparations [4]. Significant progress in the diagnosis of diseases caused by dermatophytes was made by Sabouraud in 1910, who was the first to introduce the cultivation of specimens on artificial media, emphasizing the importance of describing the cultural characteristics and morphology of fungal pathogens in the diagnosis of dermatophytosis [5]. Further progress in dermatophyte taxonomy was contributed by the introduction of nutritional physiological tests and tolerance tests as methods independent of morphology [6]. A new approach to the taxonomy of dermatophytes was given by the introduction of biological species complexes, which define species as groups of closely related populations. Within this approach, *T. rubrum* complex and *T. mentagrophytes* complex were defined, which cause the majority of human dermatophytoses [7]. A newly identified dermatophyte species from *T. mentagrophytes* complex, described in 2019, is *T. indotineae*, notable for severe infections, antifungal resistance, and global spread [8].

In recent years, there has been significant progress in dermatophyte taxonomy as a result of planned genome projects, but they do not represent the genome data of skin fungi [7,9]. The gold standard in the identification of dermatophyte species today is based on ITS (internal transcribed spacer) ribosomal RNA sequences [4]. In recent years, the “one fungus = one name” initiative has been started to assign one name to a unique ITS sequence regardless of the fungal growth phase and morphological characteristics in order to improve and facilitate the taxonomy of dermatophytes [10].

Today, in most routine laboratories in the world, the conventional laboratory identification of dermatophytes is still based primarily on the examination of macroscopic and microscopic characteristics of isolates, with the addition of physiological tests for atypical isolates, if necessary, and susceptibility testing [7,11,12,13]. Since there is no need for laboratory biomarkers as in invasive fungal disease [14], the introduction of molecular methods has significantly improved laboratory diagnosis, especially that of onychomycosis [15].

Dermatophytes are highly contagious [12,16]. Depending on their natural habitat and ecological niche, dermatophytes can be anthropophilic, geophilic, and zoophilic [16]. The majority of human dermatophytoses are caused by anthropophilic species, followed by zoophilic species. Species from all three niches are associated with human diseases and are classified based on the infection site [17]. Geophilic dermatophytes live in the soil and can occasionally cause infections in humans and animals. Zoophilic dermatophytes parasitize the skin and hair of animals but can also be transmitted to humans. Anthropophilic dermatophytes infect humans and can be transmitted from an infected person by direct or indirect contact. Anthropophilic species of dermatophytes mainly cause chronic, relatively non-inflammatory infections that are usually difficult to treat. On the other hand, geophilic and zoophilic species cause a strong host reaction, and the lesions are very inflammatory and respond well to therapy [18]. Therefore, the identification of dermatophytes at the species level is of prognostic significance and also enables the application of adequate prevention measures.

Dermatophytes are widely distributed in the environment, with an estimated prevalence of 20–25% of the the global population yearly, representing the fourth most common human disease [17,19]. Rokas and associates report that the most common fungal diseases are superficial infections caused by *Trichophyton rubrum*, *T*. *tonsurans*, *Microsporum canis*, and *Malassezia globosa*, affecting at least one billion people globally [20]. Infection with dermatophytes occurs by the transfer of arthroconidia or hyphae, or keratin material containing these fungal elements, from an infected person to a susceptible host [21]. Humans can acquire dermatophytosis when they come into direct contact with the soil or other reservoirs where structures of these fungi can be found, or the acquisition can additionally occur by contact with an infected person [22]. As strictly pathogenic agents, dermatophytes do not require previous trauma at the entry site [23]. Direct interhuman transmission is also possible given that the most common causative agents are anthropophilic dermatophytes. The transmission of anthropophilic species (*T. rubrum, T. interdigitale*) is particularly facilitated in urban, densely populated environments where people engage in numerous social activities [24]. It is observed that transmission can also be obtained by fomites, especially objects that are in close contact with humans [25]. Much less often, the infection can occur through contact with animals and soil in zoophilic (e.g., *T. verrucosum*) and geophilic species [26]. Dermatophytes can remain viable for a long time in desquamated skin cells, hair, and nails. In the external environment, they can remain viable for over six months, which significantly contributes to the prevalence of this disease and facilitates transmission indirectly from fomites [25,27]. The pattern of this infectious disease covers a wide spectrum from exposed individuals without symptoms to those with acutely inflammatory or non-inflammatory, chronic to invasive, and even life-threatening symptoms [16].

This review summarizes current information on the pathogenicity, virulence factors, and drug resistance mechanisms associated with dermatophytes.

## 2. Pathogenesis

**Figure 1:**
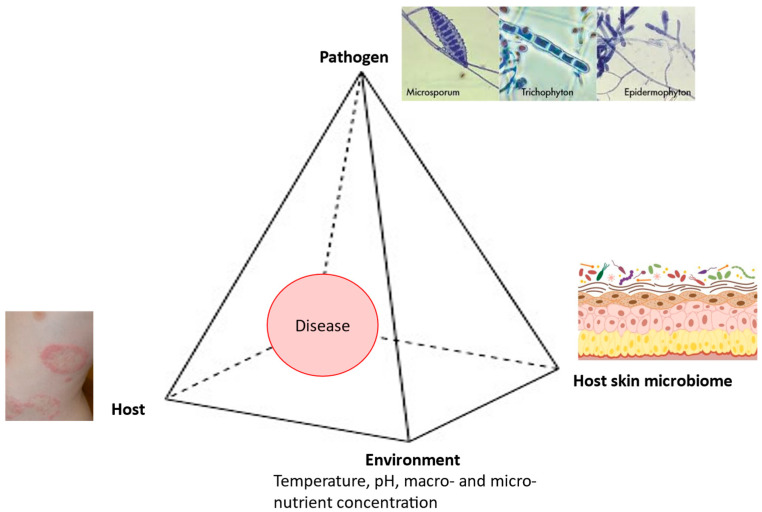
Disease pyramid in dermatophytosis.

In the pathogenesis of dermatophytoses, the fungus–host relationship is very specific, because these fungi cause diseases in immunocompetent individuals while attacking only the superficial keratin structures. Therefore, the clinical expression of dermatophytoses directly depends on both the characteristics of the causative fungi and the characteristics of the host. Geophilic and zoophilic species of dermatophytes mainly cause acute infections characterized by the rapid elimination of the causative agent through the use of adequate therapy combined with effective mechanisms of innate and acquired immunity. As a rule, anthropophilic species cause chronic infections characterized by very few or even the complete absence of symptoms, longer, and in some cases uncertain, therapeutic outcomes, as well as less effective immune responses [28]. The onset of a disease depends on the interplay between the pathogen, the host, the environment, and the host skin microbiome (Figure 1). 

## 3. Pathogen

Pathogens’ virulence represents the major factor for the successful colonization of the host tissues. It is considered that a greater number of virulence factors of these fungi are important for the occurrence of infection and the changes that occur. This fungal arsenal of virulence factors includes adhesins, secreted enzymes, the structure of the cell wall, and others.

### 3.1. Adherence and Adhesins

To avoid fungal elimination, the attachment of fungal spores to the host surface must occur rapidly within 3–4 h, followed by their germination within 24 h. Studies on experimental models have shown a time-dependent increase in the number of adhering spores, followed by germination and the invasion of the stratum corneum by hyphae growing in different directions [18]. The first studies showed that the maximum adherence of arthroconidia of fungi of the genus *Trichophyton* to keratinocytes occurs in about 3–4 h. 

The ability of *T. rubrum* to adhere to mannose and galactose of keratinocytes is attributed to carbohydrate-specific adhesions (CSA) expressed on the surface of microconidia [29]. The presence of glycoproteins containing mannan in the cell wall allows for the adherence of dermatophytes to the host cell surface, possibly in a pH-dependent manner.

Monod et al. have determined that there is a unique dipeptidyl-peptidase IV (DppIV) identified in *Trichophyton rubrum* that plays a significant role in the adherence of this fungus to keratinocytes [30].

During the adherence of *T. mentagrophytes*, the presence of fibrillar structures on the surface of the fungal cell was observed, which connect the fungal arthroconidia to the keratinocytes on the surface of the skin and nails. In the deeper layers of the skin and nails, the newly formed arthroconidia create thin and short shoots that cover the entire surface of the arthroconidia. This enables the creation of a large contact surface between the conidia and the skin or nails [31].

In *Microsporum canis*, the influence of secreted protease of the subtilisin family Sub3, metalloproteases, and dipeptidyl-peptidases on the adherence process and the early phase of invasion is examined [30].

Based on the fact that secretory aspartate proteases play a key role in the adherence of *Candida albicans* to epithelial cells, it is thought that dermatophyte secretory proteases may also facilitate or even be essential for effective adherence [32].

### 3.2. Cell Wall Structure

The fungal cell wall in dermatophytes contains mannan, a glycoprotein component, which has been demonstrated to facilitate infection by inhibiting the proliferation of keratinocytes, thus preventing shedding and the suppression of the inflammatory response. The amounts of mannan produced from *Trichophyton rubrum* are larger than in *Microsporum canis* and more effectively inhibit cell proliferation and lymphoproliferation [18].

### 3.3. Growth on Keratin Structures and Protein Degradation

Dermatophytes are primary pathogenic fungi that require the cleavage of the keratin found in the skin, hair, and nails to acquire nutrients for their metabolism [33]. 

**Figure 2:**
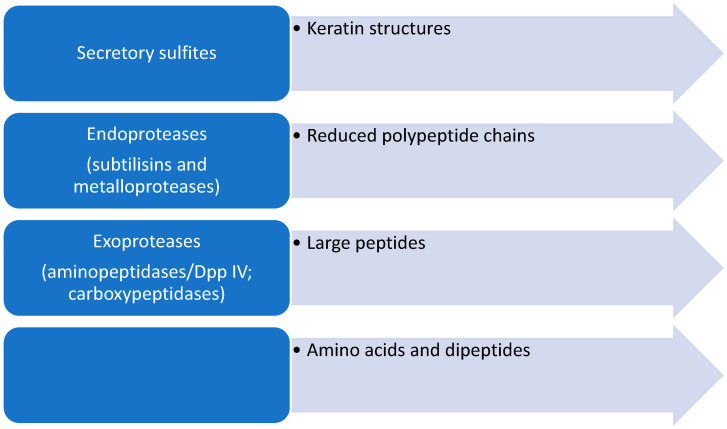
Schematic representation of the steps in the degradation of keratinous structures by dermatophytes.

Keratin-rich tissues such as the epidermis, nails, and hair contain, in addition to keratin, a network built of various cross-linked proteins such as involucrin, loricrin, and small proline-rich proteins that build the cornified cell envelope [34]. Stratum corneum proteins, especially loricrin- and proline-rich proteins, contain numerous cysteine residues that form disulfide bridges. This leads to the formation of an insoluble protein complex built from a network of cross-linked proteins. For the efficient degradation of proteins in keratin structures, it is necessary to first reduce the cystine disulfide bridges so that further secretory proteases can degrade compact keratin structures (Figure 2).

### 3.4. Reduction in Cystine Disulfide Bridges

During infection, dermatophytes and filamentous fungi excrete sulfites by the sulfite efflux pump SSU1, which have a reducing role [2]. In the presence of sulfites, disulfide bonds in keratin structures are broken, and cysteine and S-sulfocysteine are formed. Sulfitolysis is an essential step in the breakdown of complex keratin structures. In this way, reduced proteins become available for further degradation with the help of proteolytic enzymes [35].

### 3.5. Secretory Proteases of Dermatophytes

The secretion of a broad spectrum of lytic enzymes by the fungal hyphae in dermatophytes represents their most studied virulence factors, allowing fungal colonization and maintenance in the host tissue [33]. The ability to invade keratinized tissue is directly related to the enzyme production by the fungi. Dermatophytes produce numerous proteases, including collagenolytic and elastolytic enzymes, and especially keratinases, whose goal is to break down keratin into oligopeptides and amino acids that can be further absorbed and used in their metabolism [36]. Other types of enzymes can also be produced by dermatophytes to degrade host cells such as alkaline phosphatase, elastases, collagenases, lipases, nucleotidases, mucolytic enzymes, and N-acetyl-beta-glucosaminidase [19]. However, there are variations in the quantity and specificity of enzymes produced in different situations, such as dermatophytes isolated from the soil, asymptomatic animals, and symptomatic animals, with differences between the stages of the disease [22]. On the other hand, keratinase production is demonstrated by all isolates of dermatophytes since keratin is their primordial substrate [33].

Secretory proteases of dermatophytes are important virulence factors of these fungi, and a better understanding of the action and capacity of clinical or environmental dermatophytes to produce these virulence factors can help to elucidate the mechanisms behind human clinical cases. This can be essential for understanding their survival in the environment and to impact the prevention, control, and treatment of these important infectious diseases. Secretory proteases are divided into endoproteases and exoproteases [35].

Dermatophytes secrete two groups of endoproteases: subtilisins (serine proteases) and fungalysins (metalloproteases). Endoproteases break peptide bonds within polypeptides. Dermatophytes possess a genome that encodes a battery of secretory proteases similar to that of fungi of the genus *Aspergillus*. In *T. rubrum*, five MEP (genes for endometalloproteases) and seven SUB (genes for serine proteases) genes were detected [37]. These genes were also found in other dermatophytes such as SUB1-3 and MEP1-3 in *M. canis*, SUB1-7 in *Epidermophyton floccosum*, SUB 3 and 6 in *Trichophyton benhamiae*, as well as SUB genes in *T. verrrucosum* and *M. gypseum* [38,39,40,41,42].

Dermatophytic subtilisins have an average molecular weight of 30–37 kDa and are not glycosylated. Dermatophytic fungal lysins are glycoproteins with a molecular mass of 40–48 kDa that show optimum activity at pH 7–8 [35].

Exopeptidases break peptide bonds only at the N- and C-terminal ends of peptide chains. *T. rubrum* secretes two leucine aminopeptidases, Lap1 and Lap2, as well as two dipeptidyl-peptidases, DppIV and DppV. Due to the demonstrated affinity for 7-amido-4-methylcoumarin (Leu-AMC) as a substrate, Lap 1 and Lap 2 are called leucine aminopeptidases. Dipeptidyl peptidases are glycoproteins and belong to serine proteases [34]. Dermatophytes also secrete metallocarboxypeptidase (McpA), which is homologous to the human pancreas carboxypeptidase A. Also, the secretion of two serine carboxypeptidases, ScpA and ScpB, was detected in *T. rubrum* [35].

The crucial role in the regulation of protease expression during dermatophytosis for the successful establishment, development, and maintenance of dermatophyte infections in the host is the pH of the environment [43]. Initially, dermatophytes produce sensing transcription factors such as PacC and Hfs1, which allows them to adapt to acidic pH that is normally present in the skin [44]. In an acidic environment, the secretion of some dermatophyte enzymes is facilitated, such as aspartic protease Pep 1 and acetamidase [44]. Consequently, acetate and ammonia are produced, which will shift the ambient pH from acidic to alkaline, reaching values of 7.5–8.9 [43]. The shift in the ambient pH is essential because in further pathogenesis after the keratin degradation environment, the pH will shift to alkaline, and fungi will have enough time to elevate protease enzyme activity [19]. In skin and nail infections caused by dermatophytes, the raised expression of the serine protease Sub 3 and metalloprotease Lap 1 is observed in alkaline pH [19]. During the alkalinization process, dermatophytes respond by expressing enzymes that are functional at the current ambient pH values. This adaptive response is the essence of the pH regulatory system [45]. Furthermore, growth on keratin leads to the overexpression of genes encoding several proteases and membrane transporter proteins. Consequently, the presence of keratin as the carbon source and an adequate ambient pH shift are necessary to induce the expression of these genes [45].

### 3.6. Toxins

In the pathogenesis of dermatophyte infections, tissue damage can also be a result of toxin production [18]. The most investigated toxin in dermatophytes is xanthomegnin, released by *Trichophyton megninii*, *Trichophyton rubrum*, *Trichophyton violaceum*, and other dermatophytes [46]. Moreover, the production of hemolysins was observed in *Trichophyton rubrum* and *Trichophyton interdigitale*. Furthermore, some dermatophytes are observed to produce lipophilic toxins and aflatoxin-like substances that cause immunosuppressive reactions in the host [18]. 

### 3.7. Biofilm Formation

Biofilms are one of the most prevalent forms of microbial growth in nature, and many bacteria and *Candida* species are well-known etiological agents of biofilm infections [47,48]. Later, it was observed that also other yeast and filamentous fungi like dermatophytes are significant biofilm producers [48]. Biofilms are characterized by thick biomasses of an organized three-dimensional structure comprising a dense network of dormant sessile yeast and filamentous fungal cells embedded in an exopolymeric matrix. This self-produced extracellular matrix is composed of carbohydrates, proteins, and nucleic acids and is a major feature that distinguishes biofilms from planktonic cells [49]. When present in the form of biofilm, which represents highly organized communities, dermatophytes are protected from environmental stress, and the present cells exhibit intensive metabolic cooperation and cell-to-cell communication [50]. Quorum-sensing is a special form of intercellular communication, and different quorum-sensing molecules are detected in fungi, which could have an important role in biofilm formation and virulence [51]. Growth in the form of biofilm represents a biological advantage, and biofilm formation is considered a significant virulence factor in dermatophytes [52]. Many dermatophytes have been shown to form biofilms, such as the refractory form of onychomycosis—dermatophytoma, in which fungal cells are firmly attached to the nail plate and very difficult to treat [53,54].

### 3.8. Heat Shock Proteins 

Heat shock proteins (HSPs) are a conserved family of molecular chaperones that participate in multiple important biological functions in cells whose transcription responds rapidly to temperature shifts [55]. They play a crucial role in the stabilization and correct folding of nascent polypeptides, the assembling of protein complexes, and the transport and sorting of proteins into their cellular compartments [56]. Additionally, they also participate in diverse cellular functions and have a fundamental role in cell cycle control, programmed cell death control, cellular recovery from several stress conditions, and protection from subsequent insults [55]. During the infection and invasion of the host tissue, different HSPs are detected, such as the overexpression of Hsp 30, HSP 60, HSP70, HSP 78, and HSP 70 and of HSP 90, HSP-related gene hsf1, and HSPSSc1 in *T. rubrum* when grown on human nails or skin, respectively [45,56].

## 4. Host

Host susceptibility to dermatophytes primarily depends on genetic factors and the immune status, which are especially important in invasive dermatophytosis [57]. Impaired epidermal and immunological barriers are well-known favoring factors, while different patient populations have variable susceptibilities to dermatophyte infections [58,59]. Various chronic conditions predispose individuals to dermatophyte infections, such as metabolic disorders, especially diabetes, obesity, psoriasis, hyperhidrosis, and peripheral circulatory diseases [17]. Genetic factors can contribute to the increased probability of dermatophyte infections observed in patients with mutations in the gene CLEC7A and the genes of signaling pathways such as CARD9 and STAT3 [18]. Furthermore, mutations in the caspase recruitment domain-containing protein gene (CARD9) are associated with invasive dermatophytosis, and those in the HLA-DR8 haplotype are associated with onychomycosis [58,60].

Individual susceptibility factors can also predispose individuals such as alterations in sebum fatty acids, the presence of moisture or transferrin, and other inhibitors for dermatophyte growth in sweat or serum and skin, including carbon dioxide concentrations [61]. Furthermore, an older age, living in low socioeconomic conditions, close contact with animals, using antibiotics and immunosuppressive drugs, using closed shoes, excessive sweating, and contact with contaminated objects or people are also factors [61]. Additionally, the release of antimicrobial peptides at the infection site and topical steroid use can contribute to the impaired clearing of the fungi from the skin [61]. 

## 5. Environment

The environment corner refers to the host skin microenvironmental parameters and includes, among others, the temperature, pH, and micronutrient concentrations that are essential for the optimal growth of dermatophytes. 

### 5.1. Temperature

In contrast to other human pathogens, dermatophytes grow best at a temperature lower than human blood heat. In the majority of dermatophytes, the optimal temperature range was found to be 27–33 °C. For example, the optimum has been determined as 30 °C for *Trichophyton interdigitale*, 35 °C for *T. mentagrophytes,* and 25–30 °C for *Microsporum* spp. However, the ability to grow over a wide range of temperatures is a clear advantage [62]. Furthermore, geographical regions can also affect the prevalence of dermatophytosis, so higher observed values were found in areas with high temperatures and humidities, such as tropical and sub-tropical regions [63]. 

### 5.2. pH

Dermatophytes are not exacting in their pH requirements, and a pH range of 4–10 is compatible with the growth of most species, with the optimum being slightly lower than neutrality. The pH range of 7.2–8.0 is favorable for the production of proteolytic enzymes (keratinases) by keratinophilic fungi, which are necessary for their growth [64]. Once established, dermatophytes seek nutrients for growth and respond to the ambient pH by derepressing genes encoding proteins and enzymes that have optimum activity at acidic pH values, such as adhesins, lipases, phosphatases, DNAses, and keratinolytic proteases, among many others. The acidic pH of the skin is optimal for these enzymes, allowing for the adherence and penetration of the host tissue, the uptake of nutrients, and survival against host defense mechanisms. Some studies found that in acidic environments, there is the growth inhibition of dermatophytes and other keratinophilic fungi, but environments that are weakly acidic to neutral or alkaline are optimal for their growth [64].

### 5.3. Macro- and MicroNutrient Concentration

Fungi are heterotrophic and absorb nutrients from the environment through branching mycelia that have a high surface-area-to-volume ratio, which allows for the efficient absorption of nutrients [65]. Dermatophytes are well adapted to the skin environment and carbohydrate-poor but protein-rich conditions that are present in the two outer layers of the skin. Unlike other fungi, during evolution, dermatophytes have adapted to keratin and associated proteins in the skin, with developed efficient systems for its digestion and utilization [66]. However, dermatophytes are still able to grow on various carbohydrates such as glucose, mannose, fructose and galactose, cellobiose, and trehalose. Amino acids and oligopeptides are the main sources of energy and carbon, and they provide nitrogen for fungal growth [2]. Furthermore, iron and zinc, in addition to the essential elements potassium, phosphorus, magnesium, and sulfur, are required for the optimal growth of dermatophytes [18].

## 6. Host Microbiome

The efficient establishment of the dermatophyte as the primary pathogenic fungus is influenced by the host microbiota skin community structure. This unique ecosystem is dominantly formed by various bacteria, while the number and diversity of fungi are significantly lower [67,68]. The role of this environmental niche is in the first line of defense against various invading microorganisms [69]. However, human skin colonization by fungi is hard to establish since the temperature is unfavorably high, the skin is relatively dry, and nutrient availability is not only limited but the competition for them is high [69]. Furthermore, an acidic environment in the skin interferes with colonization due to a combination of molecules derived from glands, epidermal cells, and resident microbiota [57].

## 7. Dermatophytosis

Dermatophytes are causative agents of the cutaneous skin disease called dermatophytosis, ringworm, or tinea which are the most prevalent fungal infections affecting the skin and its adnexa both in immunocompetent and immunocompromised individuals [70]. The disease affects parts of the human body that are rich in keratin such as skin, hair, and nails. Only *Trichophyton* is capable of affecting all three structures, while *Microsporum* targets skin and hair, and *Epidermophyton* targets skin and nails based on an affinity for different classes of keratin [17].

Dermatophytes do not represent a part of normal skin microbiota, but still, 30–70% of the human population are colonized, which is related to asthma, allergy, or eczema in susceptible hosts [46]. A typical dermatophytic skin lesion is ring-shaped with a clear center and inflammatory edge, erythematous, and itchy, while in onychomycosis, nails become thicker, discolored, and separated from the nail bed, and sometimes, white spots and dystrophy may also occur [17].

### Deep Dermatophytosis

Even though dermatophytes affect the stratum corneum, they can rarely cause more severe, extensive, and invasive infections, such as deep or extensive dermatophytosis and Majocchi’s granuloma [71]. The invasion and dissemination of dermatophytes are reported in various underlying conditions such as patients with leukemia or lymphoma, diabetes, hepatitis B and C-related cirrhosis, atopic dermatitis, hemodialysis for renal failure, alcoholic liver disease, congenital adrenal hyperplasia, and Cushing disease, as well as patients on immunosuppressants/modulators for systemic lupus erythematosus, psoriasis, rheumatoid arthritis, Behcet’s disease, autoimmune hepatitis, and myasthenia gravis [72].

## 8. Immune Response

The immune response to fungi involves protective mechanisms that are detected early in the evolution in multicellular organisms (innate immunity) as well as mechanisms that are specifically induced during infection and disease (acquired immunity) [18,73].

### 8.1. Innate Immune Response

The immune response to dermatophytes begins with the interaction of the fungi with cells of the stratum corneum. In the presence of dermatophyte antigens, such as trichophytin, keratinocytes release IL-8 which leads to the accumulation of neutrophils in the stratum corneum [74]. Therefore, keratinocytes not only play a role in the formation of a mechanical barrier but also play a significant role in initiating the skin inflammatory reaction [75]. In the initial stages of infections, fungi in the group of dermatophytes first colonize the appropriate skin region and subsequently stimulate keratinocytes to produce additional cytokines. In response to their release, an inflammatory response is initiated, and in tissue that is affected by fungi, the accumulation of neutrophils and other leukocytes starts [55]. Which specific cytokines will be released in response to the dermatophyte invasion of the host tissue is associated with fungal species. Hau and colleagues have shown that anthropophilic species will induce lower amounts of cytokines. For example, *Trichophyton tonsurans* will mainly induce the secretion of IL-8, IL-6, and IL-1b by keratinocytes [76]. In contrast, a much broader spectrum of secreted cytokines is observed in dermatophytosis caused by zoophilic species such as IL-8, IL-6, IL-1b, IL-10, IL-2, IL-15, and TGF-b. This will result in more clinically pronounced lesions as a result of a much higher number of inflammatory cells in the infection site, which is responsible for fungal clearance from affected tissue, healing, and tissue remodeling [76].

Polymorphonuclear leukocytes (PMNL) and monocytes play a significant role in the body’s defense against fungi. Phagocytosis and respiratory burst are the two main mechanisms by which PMNL kill fungi. Macrophages phagocytize *T. rubrum* conidia, while exoantigens or mannans of this fungus can inhibit this process [77]. Receptors on macrophages, as well as on other host innate immune cells, are responsible for the initial recognition of dermatophytes and the further activation of the adaptive immune response to control the infection. Phagocytosed conidia of *T. rubrum* affect the downstream regulation of MHC II (major histocompatibility complex) molecules as well as the expression of costimulatory molecules and induce the production of the strong anti-inflammatory cytokine IL-10. Also, within the macrophages, the differentiation of conidia into hyphae can occur, which can lead to the death of the macrophages themselves. In this way, fungal cells inhibit the function of macrophages and induce suppressive cytokines to avoid the immune response [78].

The immune system has also developed a pathogen surveillance system, the so-called pattern recognition receptors that recognize frequently encountered structures of microorganism products. Toll-like receptors (TLR) also belong to this group. It has been observed that TLR-2-dependent mechanisms induced by certain microorganisms (probably also *T. rubrum*) lead to the avoidance or inhibition of the immune response [79].

Dectin-1, a β-glucan receptor primarily found on macrophages and dendritic cells, is a small type II transmembrane receptor containing lectin-like carbohydrate recognition domains that recognize β 1,3- and β 1,6-linked glucans as well as intact cells’ yeast. Dectin-1 mediates the cellular response to yeast or fungal conidia by inducing the production of proinflammatory cytokines [80].

Another carbohydrate receptor, dectin-2, was discovered, which binds dominantly to the hyphae of *T. rubrum* as well as to the hyphae of other fungal species and leads to the activation of macrophages [81].

### 8.2. Acquired Immune Response

Numerous studies have shown that the humoral immune response to dermatophytes is not protective [77]. However, antibodies can be detected in infected animals and humans. High concentrations of IgE and IgG4 antibodies were detected in patients with chronic dermatophytosis. On the other hand, antibody concentrations are low in people with a positive delayed hypersensitivity skin test. A positive early hypersensitivity test is often seen in individuals with chronic dermatophytosis [74]. However, the production of antibodies and complement activation is also important to control dermatophytes [45]. In patients with dermatophyte infections, serum analysis demonstrated high levels of Th2 cytokines, such as IL-4, IL-5, and IgE [76,82,83].

The characteristic cell-mediated immune response to fungi represents a late type of hypersensitivity in which the ultimate effector cells are activated macrophages. The activation of macrophages occurs due to the production of interferon (IFN-γ) by CD4+ T lymphocytes. This response is characterized by induration at the injection site due to cell recruitment to the skin and associated fibrin deposition. A positive delayed hypersensitivity reaction is associated with a low titer of IgG antibodies directed against *Trichophyton* fungi antigens and the absence of IgE or IgG4 antibodies [75]. In controlling dermatophytoses, the induction of adaptive immunity by innate cells triggers both Th1 and Th17 responses [76,84]. Heinen and associates found that the Th17 response increases the infiltration of neutrophils in the infection site, in turn promoting the activation of epithelial cells to produce chemotactic molecules and antimicrobial peptides [85]. Observed differences in the immune response may also be the result of infection with different dermatophyte species, as well as a reflection of the immunological status of the host [76,86].

## 9. Antifungal Resistance 

The treatment of infections caused by dermatophytes is limited to a few antifungal drugs such as the azole compounds itraconazole, ketoconazole, fluconazole, then terbinafine, and griseofulvin [87]. High keratin adherence is reported in terbinafine, itraconazole, and ketoconazole, and they are considered as the treatment of choice for the majority of dermatophytosis patients [88]. Antifungal drug resistance in dermatophytes was first reported shortly after the turn of the millennium and has today been reported in *Trichophyton* and occasionally in *Microsporum*, but not in *Epidermophyton* species.

The first cases of terbinafine-resistant *T. rubrum* were reported at the beginning of the new millennium [89,90], and since then, antifungal drug resistance in dermatophytes has continued to extend [87]. In the last two decades, cases of treatment failure due to terbinafine resistance in patients with tinea pedis, onychomycosis, and tinea corporis caused by *T. rubrum* and *T. interdigitale* have been reported increasingly often in Europe and Asia [91,92,93,94,95]. Non-synonymous point mutations in the Squalene Epoxidase (SQLE) gene of *T. rubrum* and *T. interdigitale* were found to be fully responsible for terbinafine resistance [96]. As a result of mutations in the squalene epoxidase gene, the terbinafine cannot inhibit the enzyme anymore, so there is no reduction in ergosterol synthesis. Thus, cell death cannot occur (no fungicidal effect), giving rise to resistance. [97]. The amino acid substitutions in squalene epoxidase were demonstrated in *T. rubrum* such as L393F and F397L, and the use of molecular sequencing remains the best way to characterize strains that are resistant to terbinafine [97]. In Europe, terbinafine resistance is still low, in contrast with the high prevalence in India, where is estimated that up to 70% of *Trichophyton mentagrophytes* isolates causing tinea cruris and tinea corporis are resistant to terbinafine and are even suggested to be distinguished as *T. indotineae* [98,99]. The emergence of the pathogen has been linked to the abuse of topical steroid-containing fixed-dose combination creams and erratic treatment with antifungal agents that are so very prevalent in India but represent a global threat since *T. indotineae* has rapidly become ubiquitous across the globe [94]. 

Resistance to azoles is also continuing to be reported and is associated with the overexpression of two genes (MDR2 and MDR 3) encoding a multidrug transporter of the ABC family, giving rise to multidrug efflux outside the cell [96]. Additionally, a multicenter study conducted by Kong et al. found that the double substitution F397L/A448T was associated with higher MIC values for triazoles in addition to MICs > 16 µg/mL for terbinafine that could develop cross-resistances to azoles and allylamines [100]. A recent study showed that a pleiotropic transporter, the major facilitator superfamily (MFS1), also seems to be implicated in azole resistance. It was found that the suppression of MFS1 in *T. benhamiae* increases sensitivity to fluconazole and miconazole, while no effect was seen for chloramphenicol [101]. Reports of cross-resistance to azoles and allylamines are becoming more frequent, but promising results were described with newel triazoles, luliconazole, and lanaconazole. Compared to fluconazole and itraconazole, they showed higher efficacy and therefore could represent a good alternative in the treatment of recalcitrant dermatophytosis. Still, their use in the real-life treatment of dermatophytosis is limited [97]. 

Even though resistance in *Trichophyton* spp. is increasingly reported worldwide, antifungal susceptibility testing is still not routinely investigated in many mycological laboratories worldwide [13,99,102]. However, the highest rates are observed in India (36% and 68% for terbinafine (MIC ≥ 4 mg/L) and fluconazole (MICs ≥ 16 mg/L), respectively) and involve the spread of a unique clade related to the *Trichophyton mentagrophytes*/*Trichophyton interdigitale* complex. The European Committee on Antimicrobial Susceptibility Testing (EUCAST) has developed a dermatophyte antifungal susceptibility testing method (E.Def 9.3.2) based on the microdilution method that has been validated for terbinafine, voriconazole, itraconazole, and amorolfine against *T. rubrum* and *T. interdigitale*.

## 10. Conclusions and Future Perspectives

The investigation of host–pathogen interactions is essential for developing a more complete understanding of the mechanisms underlying dermatophyte pathogenesis and host responses to inform the use of diagnostics methods and antifungal therapeutics in order to minimize the high fungal burden caused by dermatophytes. The need for a deeper understanding of dermatophyte–host interaction is necessary owing to a constant increase in dermatophytosis and growing evidence of susceptible hosts with severe clinical presentations. The constant surveillance of antifungal susceptibilities in clinical isolates of dermatophytes is necessary to control the spread or resistance.
